# Operation for Obstruction of the Bowel

**Published:** 1882-04

**Authors:** W. A. J. Anderson

**Affiliations:** Oxford, Ga.


### OPERATION FOR OBSTRUCTION OF THE BOWEL.

By W. A. J. ANDERSON, M D., Oxford, Ga.

Without attempting to give the symptoms of obstruction of the bowel, whether hernia, invagination, volvulus, inflammation, or other cause; or without dilating upon the apparent improbability of invagination, or of the twisting or knotting of the bowel in view of its mesenteric attachments, I shall at once proceed to the report of a case of obstruction, and operation therefor, which pretty fairly represents three or four other cases similarly operated on.

Some time in October, 1877 (or '78) I was called to a robust, muscular negro man, in Rockdale county, at Glenn's mills (on the premises of Dr. Glenn). I learned he had been in great agony for eight or ten days, and had been treated by Dr. Stewart, of Conyers, for obstruction of the bowel, with emetics and cathartics, for several days, without any permanent relief. I remained with him an hour or two, and found the pains returned at regular intervals, as in labor pains. The abdomen was greatly distended and very tender over all parts of it; great prostration, weak pulse, stercoraceous vomiting, which had been kept up for several days. I prescribed injections of strained gruel, and gave him a small quantity of rye whisky, which he said he "natally honed for," (longed for). The next day Dr. J. A. Stewart met me, and after a short talk we concluded to perform the abdominal section. We chloroformed him, and according to his urgent request, we "let out his bowels." I cut through the umbilicus on the linea alba, and coming to the gut, found it sound. There was no prominence or point indicating the intususception. Dr. Stewart then put in the director, and I made an incision two or three inches long. Placing my left hand firmly over the bowels, I handed the bistoury to Dr. Stewart and requested him to extend the incision an inch or two further. He did so; and out came the bowels to the height of several inches. The assistant having let up on the chloroform, and the patient, seeing his entrails, made a great effort to rise, which caused them to protrude still further. The sponge being instantly reapplied, he was again quickly under the chloroform, when Dr. Stewart proceeded to examine the bowels, finding a clear case of intususception of about six or eight inches. In the meantime the chloroform having been withdrawn, and the patient "coming to," he instantly placed his hand below the incision and exclaimed, "Thank God, it's gone past," a loud growling of guts coming on simultaneously. Now, the point I wish to call attention to is this: Was it letting out the bowels, causing the remaining bowels to rotate, or untwist, or "come out of the kink;" or what?

We then stitched up the wound with strong silk thread, and applied strong adhesive strips. There was no return of pain; almost perfect ease succeeded the operation, with copious evacuation of fæces. I visited him the next day and found him sleeping soundly. I awoke him and gave him some whisky (as he still "natally honed for it,") and allowed him some sweet potatoes, for which he had a similar penchant.

The after treatment was conducted by Dr. Stewart, without my seeing him again.

